# Knowledge, attitude, and practice (KAP) of medical staff toward behavioral and psychological symptoms of dementia (BPSD) in medical institutions in Xinjiang Uygur Autonomous Region

**DOI:** 10.3389/fpsyt.2026.1825862

**Published:** 2026-07-17

**Authors:** Lizhen Wu, Anqi Zhang, Wenhui Zhang, Peipei Jiang, Xiaojie Ma, Maimaitirexiati Tuerxun

**Affiliations:** The Fourth People’s Hospital of Urumqi, Urumqi, China

**Keywords:** BPSD, dementia, KAP, psychiatry, Xinjiang Uygur Autonomous Region

## Abstract

**Background:**

This study aimed to assess the knowledge, attitude, and practice (KAP) of medical staff in Xinjiang Uygur Autonomous Region (Hereinafter referred to as Xinjiang) concerning behavioral and psychological symptoms of dementia (BPSD) and to identify associated influencing factors.

**Methods:**

A cross-sectional survey was conducted from December 2024 to January 2025 across 13 medical institutions in Xinjiang, utilizing the Questionnaire Star platform for data collection. The survey instrument comprised demographic information and self-reported KAP scores related to BPSD.

**Results:**

Among 713 valid responses, after adjustment, psychiatric medical staff had significantly higher knowledge scores than non-psychiatric medical staff (β = 2.548, P < 0.001), with no significant differences in attitude or practice scores. Within the psychiatric cohort, knowledge scores demonstrated a negative correlation with age (r = -0.131, *P* < 0.001), while attitude scores were inversely associated with professional title (r = -0.137, *P* < 0.001).

**Conclusion:**

Young psychiatric medical staff have a better understanding of BPSD knowledge. Targeted professional training is essential.

## 1 Introduction

Dementia, as a type of progressive neurodegenerative disease closely related to aging, is posing an increasingly severe public health challenge amidst the global aging population, with its prevalence continuing to rise (1). Currently, approximately 55 million people worldwide suffer from dementia (2), and it is projected that the number of patients will increase to 153 million by 2050 (3). As China’s aging process accelerates, the burden of dementia is also rapidly rising, accounting for about 25% of the global dementia population (4). Studies on the regional distribution of dementia prevalence in China reveal a pattern where the north and west regions have higher rates, with Xinjiang Uygur Autonomous Region (Hereinafter referred to as Xinjiang) being particularly prominent (5). Behavioral and psychological symptoms of dementia (BPSD) are core non-cognitive symptoms of dementia, encompassing apathy, agitation, and delusions. They affect approximately 60%-96.4% of dementia patients over the disease course (6, 7) significantly reducing quality of life, accelerating functional decline, increasing hospitalization risk, and imposing a considerable burden on healthcare systems, the economy, and caregivers (8).

Medical staff serve a critical role in the diagnosis and management of dementia, where their knowledge, attitude, and practice (KAP) concerning BPSD significantly impact symptom identification and therapeutic efficacy (9). However, existing literature still lacks sufficient attention to the key aspect of dementia prevention and treatment—the KAP of medical professionals. Unlike studies on dementia-related KAP in other regions of China, such as Beijing and Macao (10–12), this study focuses on Xinjiang, where the prevalence of dementia is relatively high.

## 2 Materials and methods

### 2.1 Study participants

A cross-sectional survey was conducted among medical staff from 13 medical institutions in Xinjiang between December 2024 and January 2025, using convenience sampling approach. The sample encompassed public primary, secondary, and tertiary hospitals, community health service centers, and private hospitals to ensure representativeness. The inclusion criteria were practicing physicians and registered nurses who were employed, while administrative staff, interns, and medical students were excluded. Based on the capacity of participating institutions, our goal was to recruit at least 600 participants to ensure sufficient statistical power. A total of 730 questionnaires were collected, of which 713 were valid, with a valid response rate of 97.7%. Ethical approval for this study was obtained from the Ethics Committee of the Fourth People’s Hospital of Urumqi (Approval No.: 2023-014-01).

### 2.2 Method

#### 2.2.1 Survey instrument

The KAP theoretical framework was used. The framework assumes that knowledge is the basis for attitude change, and that attitude is the forerunner of behavioral practice, thus constructing the core dimensions of the questionnaire. Combined with the recommendations of the “Guidelines for the Prevention and Treatment of Alzheimer’s Disease in China” (2021 Edition) (13), ensure that the content of the questionnaire complies with the current national professional standards, especially the management requirements for BPSD. The questionnaire consists of 4 parts (1) demographic characteristics (9 items); (2) Knowledge assessment (12 questions), using a three-point scoring system (know = 2 points, know a little = 1 point, don’t know = 0 points), with a total score range of 0–22 points. To ensure the data quality, a validity test item (K4) was embedded. For example, one item stated: “BPSD in people with dementia often occurs in infants and young children.” Respondents who chose any answer other than “don’t know” were considered careless. The K4 item was excluded from the total score calculation; (3) Attitude assessment (8 questions), using a five-point special scale (very positive = 4 points, positive = 3 points, neutral = 2 points, negative = 1 point, very negative = 0 points), with a total score range of 0–32 points; and (4) Practice assessment (7 questions), with the same rating as the Attitude assessment, with a total score range of 0–28 points. The questionnaire questions were developed based on two validated tools from published studies and combined with clinical practice (10, 14). At the same time, it has been modified to adapt to the specific situation of BPSD management in Xinjiang. The initial questionnaire was repeatedly revised by a multidisciplinary expert panel composed of a neurologist and two geriatricians and a clinical nurse., a psychiatrist, and a public health specialist. The pilot test (n = 100) showed excellent psychometric properties (Cronbach’s α coefficients of 0.946, 0.939, and 0.964 for the knowledge, attitude, and practice subscales of the questionnaire, respectively, and an overall KMO value of 0.962). The survey was conducted at 13 medical institutions through an online questionnaire platform between December 2024 and January 2025.

#### 2.2.2 Research methodology

The study utilized the “Wenjuanxing” online platform for survey distribution. Prior to data collection, informed consent was obtained electronically through Wenjuanxing, which features mandatory consent checkboxes and logical checks to prevent missing data.

#### 2.2.3 Statistical analysis

Data analysis was conducted using SPSS 26.0 statistical software. Enumeration data were expressed as the number of cases (percentage) [n (%)], and the chi-square test was used for inter-group comparison. Measurement data were first tested for normality. Measurement data conforming to a normal distribution were expressed as mean ± standard deviation (x ± s), and independent sample t-tests were used for comparison between two groups; measurement data not conforming to a normal distribution were expressed as M (Q1, Q3), and the Mann-Whitney U test was used for comparison between two groups. Multiple linear regression analysis was performed with knowledge, attitude, and practice scores as dependent variables, adjusting for confounding factors such as age, sex, education level, professional title, and years of work experience. Spearman’s rank correlation was used for correlation analysis, with a significance level of α = 0.05. *P* < 0.05 was considered statistically significant.

## 3 Results

### 3.1 Comparison of KAP of BPSD between psychiatric medical staff and non-psychiatric medical staff

This study enrolled 713 medical staff, including 518 (72.7%) from psychiatric departments and 195 (27.3%) from non-psychiatric departments. The cohort comprised 589 (82.6%) female and 124 (17.4%) male participants. Age distribution analysis revealed that 277 (38.9%) were aged 31–40 years, 246 (34.5%) were ≥ 41 years, and 190 (26.6%) were ≤ 30 years, with the 31–40 age group representing the largest proportion (*P* < 0.01). Regarding educational attainment, 623 (87.4%) held a bachelor’s degree or lower, while 90 (12.6%) possessed a graduate degree or higher. In terms of professional title (a qualification ranking system in China’s health sector), 360 (50.5%) held intermediate and above professional titles, 300 (42.1%) were junior professional title, and 53 (7.4%) had no professional title. Working years distribution showed that 428 (60.0%) had ≥ 10 years of experience, whereas 285 (40.0%) had < 10 years.

After adjusting for confounding factors such as age, sex, education level, professional title, and working years, psychiatric medical staff scored higher in knowledge than non-psychiatric medical staff (β = 2.548, *P* < 0.001). There were no statistically significant differences in attitude scores and practice scores between psychiatric medical staff and non-psychiatric medical staff (*P* > 0.05). Detailed results are presented in **Tables 1**–**4**.

**Table 1:** Comparison of KAP of BPSD between psychiatric medical staff and non-psychiatric medical staff.

Variables	Total, (N = 713) No, (%)	Psychiatric medical staff, (N = 518) No, (%)	Non-psychiatric medical staff (N = 195) No, (%)	Z/X^2^	*P*
Sex				3.213	0.073
Male	124 (17.39%)	82 (15.83%)	42 (21.54%)		
Female	589 (82.61%)	436 (84.17%)	153 (78.46%)		
Age				31.619	<0.001
≤30	190 (26.65%)	153 (29.54%)	37 (18.97%)		
31-40	277 (38.85%)	218 (42.08%)	59 (30.26%)		
≥41	246 (34.50%)	147 (28.38%)	99 (50.77%)		
Education level				2.610	0.106
Bachelor’s degree or below	623 (87.38%)	459 (88.61%)	164 (84.10%)		
Graduate degree or above	90 (12.62%)	59 (11.39%)	31 (15.90%)		
Professional title				19.948	<0.001
No professional title	53 (7.43%)	30 (5.79%)	23 (11.79%)		
Junior professional title	300 (42.08%)	242 (46.72%)	58 (29.74%)		
Intermediate and above professional titles	360 (50.49%)	246 (47.49%)	114 (58.46%)		
Working years				3.524	0.060
<10 years	285 (39.97%)	218 (42.08%)	67 (34.36%)		
≥10 years	428 (60.03%)	300 (57.92%)	128 (65.64%)		
Knowledge	19 (13,23)	21 (14,23)	16 (12,21)	35.737	<0.001
Attitude	24 (20,24)	24 (21,25)	23 (19,24)	11.302	0.001
Practice	21 (18,21)	21 (19,21)	21 (16,21)	6.133	0.013

BPSD, behavioral and psychological symptoms of dementia; KAP, Knowledge, Attitude, Practice.

Categorical variables: n (%), analyzed by Pearson χ² test.

Continuous variables: median (Q1, Q3), analyzed by the Mann–Whitney U test.

**Table 2:** Multiple linear regression analysis for Knowledge.

Variable	β	95%CI	*P*
Psychiatric medical staff	2.548	1.612-3.483	<0.001
Age (≤30)	-0.747	-1.519-0.025	0.058
Sex (Female)	0.200	-0.886-1.286	0.718
Education level (Graduate degree or above)	-0.709	-1.982-0.564	0.274
Professional title (No title)	0.487	-0.369-1.342	0.264
Working years (≥10 years)	-0.744	-1.988-0.500	0.241

Group: Psychiatric medical staff=1, Non-psychiatric medical staff=2.

Age group: ≤30 = 1, 31–40 = 2, ≥41 = 3.

Sex: Male=1, Female=2.

Education level: Bachelor’s degree or below =1, Graduate degree or above =2.

Professional title: No professional title =1, Junior professional title =2, Intermediate and above professional titles =3.

Working years:<10 years=1, ≥10 years=2.

**Table 3:** Multiple linear regression analysis for Attitude.

Variable	β	95%CI	*P*
Psychiatric medical staff	0.836	-0.048-1.720	0.064
Age (≤30)	-0.404	-1.134-0.325	0.277
Sex (Female)	-0.464	-1.489-0.562	0.375
Education level (Graduate degree or above)	0.219	-0.984-1.421	0.721
Professional title (No title)	-0.379	-1.187-0.429	0.358
Working years (≥10 years)	-0.277	-1.452-0.899	0.644

Group: Psychiatric medical staff=1, Non-psychiatric medical staff=2.

Age group: ≤30 = 1, 31–40 = 2, ≥41 = 3.

Sex: Male=1, Female=2.

Education level: Bachelor’s degree or below =1, Graduate degree or above =2.

Professional title: No professional title =1, Junior professional title =2, Intermediate and above professional titles =3.

Working years:<10 years=1, ≥10 years=2.

**Table 4:** Multiple linear regression analysis for Practice.

Variable	β	95%CI	*P*
Psychiatric medical staff	0.688	-0.157-1.533	0.110
Age (≤30)	-0.491	-1.189-0.206	0.167
Sex (Female)	-0.270	-1.251-0.711	0.590
Education level (Graduate degree or above)	0.249	-0.901-1.399	0.671
Professional title (No title)	-0.060	-0.832-0.713	0.880
Working years (≥10 years)	-0.174	-1.297-0.950	0.762

Group: Psychiatric medical staff=1, Non-psychiatric medical staff=2.

Age group: ≤30 = 1, 31–40 = 2, ≥41 = 3.

Sex: Male=1, Female=2.

Education level: Bachelor’s degree or below =1, Graduate degree or above =2.

Professional title: No professional title =1, Junior professional title =2, Intermediate and above professional titles =3.

Working years: <10 years=1, ≥10 years=2.

### 3.2 Comparison of KAP of BPSD between psychiatrists and psychiatric nurses

The study enrolled 518 psychiatric medical staff, including 244 psychiatrists (47.1%) and 274 psychiatric nurses (52.9%). A significant sex disparity was observed between the two groups, with a higher proportion of males among psychiatrists (29.92%) compared to psychiatric nurses (3.28%) (χ² = 68.710, *P* < 0.001), while females were more prevalent among psychiatric nurses (96.72%) than psychiatrists (70.08%). Age distribution analysis revealed that 42.62% of psychiatrists were over 41 years old, whereas the majority of psychiatric nurses (46.35%) were aged 31–40 years, demonstrating a statistically significant difference in age structure between the groups (χ² = 49.458, *P* < 0.001). Regarding educational background, psychiatrists exhibited a significantly higher proportion of postgraduate qualifications (23.36%) compared to psychiatric nurses (0.73%) (χ² = 65.494, *P* < 0.001). However, no significant differences were observed in professional titles between the two groups (*P* > 0.05). Detailed findings are presented in **Table 5**.

**Table 5:** Comparison of KAP of BPSD between psychiatrists and psychiatric nurses.

Variables	Psychiatrist (n=244) No, (%)	Psychiatric nurse (n=274) No, (%)	Z/X^2^	*P*
Sex			68.710	<0.001
Male	73 (29.92%)	9 (3.28%)		
Female	171 (70.08%)	265 (96.72%)		
Age			49.458	<0.001
≤30	49 (20.08%)	104 (37.96%)		
31-40	91 (37.30%)	127 (46.35%)		
≥41	104 (42.62%)	43 (15.69%)		
Education level			65.494	<0.001
Bachelor’s degree or below	187 (76.64%)	272 (99.27%)		
Graduate degree or above	57 (23.36%)	2 (0.73%)		
Professional title			53.308	<0.001
No professional title	16 (6.56%)	14 (5.11%)		
Junior professional title	73 (29.92%)	169 (61.68%)		
Intermediate and above professional titles	155 (63.52%)	91 (33.21%)		
Working years			7.823	0.005
<10 years	87 (35.66%)	131 (47.81)%		
≥10 years	157 (64.34%)	143 (52.19%)		
Knowledge	21 (16,23)	20 (14,24)	-0.598	0.550
attitude	24 (21,25)	24 (21,25)	-0.021	0.983
Practice	21 (19,21)	21 (19,22)	-0.732	0.464

BPSD, behavioral and psychological symptoms of dementia; KAP, Knowledge, Attitude, Practice.

Categorical variables: n (%), analyzed by Pearson χ² test.

Continuous variables: median (Q1, Q3), analyzed by the Mann–Whitney U test.

### 3.3 Correlation analysis of KAP scores regarding BPSD among psychiatric medical staff

The results showed that no statistically significant associations between KAP scores and occupational role (physician vs. nurse), sex, or education level (*P* > 0.05). Weak but statistically significant negative correlations were observed between KAP scores and age, professional title, and working years (*P* < 0.05). Detailed findings are presented in **Table 6**.

**Table 6:** Correlation analysis of KAP scores regarding BPSD among psychiatric medical staff .

Variables	Knowledge		Attitude		Practice	
	r	*P*	r	*P*	r	*P*
Sex	-0.054	0.219	-0.041	0.347	-0.036	0.413
Age (≤30)	-0.131	<0.001	-0.098	0.027	-0.107	0.015
Education level (Bachelor’s degree or below)	-0.01	0.822	-0.012	0.788	0.015	0.729
Professional title (No title)	-0.092	0.037	-0.137	<0.001	-0.092	0.036
Working years (<10 years)	-0.111	0.012	-0.109	0.013	-0.088	0.045

Sex: Male=1, Female=2.

Age group: ≤30 = 1, 31–40 = 2, ≥41 = 3.

Education level: Bachelor’s degree or below =1, Graduate degree or above =2.

Professional title: No professional title =1, Junior professional title =2, Intermediate and above professional titles =3.

Working years:<10 years=1, ≥10 years=2.

### 3.4 Tables

## 4 Discussion

After adjusting for confounders, including age, sex, education, professional title, and working years, psychiatric medical staff demonstrated significantly higher knowledge scores regarding BPSD than their non-psychiatric counterparts, yet no significant differences were observed in attitude or practice scores. This finding contrasts with existing literature. For instance, Baoyu Chen et al. reported that neurologists exhibited better BPSD symptom perception than psychiatrists (15), while Xianghui Liu et al. found that non-dementia specialists possess adequate dementia-related knowledge after excluding psychiatrists and neurologists (11). These discrepancies may be attributed to differences in study population sampling strategies. In response to the increasingly severe challenge of dementia, China launched the *“National Action Plan for Addressing Dementia in the Elderly (2024-2030)”* (16) and the *“National Collaborative Network for Full-Course Services of Dementia in the Elderly”* (17) in 2024. The policy requires that by 2030, the public awareness rate of dementia prevention and control knowledge should exceed 80%, and the cognitive screening rate for people aged 65 and above should also exceed 80%. The evaluation and management of BPSD require the integration of multidisciplinary medical resources and the collaborative participation of communities and families (18), However, it is worth noting that due to the vast territory and sparse population in some areas of Xinjiang, the health service radius is relatively large, and it is difficult to achieve the standard of comprehensive coverage of basic medical and health services for residents in these areas (19, 20). Therefore, this study included medical staff from different professions and institutional levels to evaluate their KAP regarding BPSD, identify obstacles and promoting factors in its management, and provide a scientific basis for developing targeted training programs and service optimization strategies in Xinjiang, thereby facilitating the achievement of national policy goals by 2030.

This study found significant demographic differences (*P* < 0.001) between psychiatrists and psychiatric nurses in terms of sex, age, educational level, and professional title, but the overall KAP level of BPSD between the two was comparable. The observed contrast between population heterogeneity and KAP homogeneity may be attributed to the standardized, team-based nature of BPSD management. Within the multidisciplinary team (MDT) framework, psychiatrists are primarily responsible for diagnostic assessment and medication treatment (21), while psychiatric nursing primarily conduct symptom assessments, implement non-pharmacological interventions, and provide nursing assistance (22). As frontline practitioners in BPSD management, psychiatric nurses typically detect symptoms earlier and carry out non-pharmacological interventions more frequently (23). This “high-frequency contact instant response” work environment may be associated with psychiatric nurses acquiring the same BPSD management skills as doctors through experiential learning. In addition, the implementation of continuing medical education policies can effectively and continuously enhance the capabilities of professional and technical personnel (24). This suggests that psychiatric nursing training may be as critical as clinical practice in BPSD management (25). These findings suggest that factors such as training and the work environment may be more strongly associated with BPSD management outcomes than individual professional roles (26), highlighting the critical need for strengthening interdisciplinary collaboration (22).

Correlation analysis revealed that age, professional title, and working years were negatively correlated with all dimensions of BPSD-related KAP among psychiatric medical staff (all *P* < 0.05). However, the correlation coefficients were very small (all |r| < 0.20), indicating limited practical significance. Therefore, the following interpretation should be considered hypothesis-generating rather than conclusive. Although previous studies have confirmed a positive association between professional seniority and BPSD care competence (10), our findings appear to contradict this. A plausible explanation is that BPSD has only gained increasing attention and dissemination in Xinjiang over the past decade. Consequently, senior psychiatric medical staff who completed their training before this period may have had limited exposure to BPSD-specific knowledge, whereas younger staff are more likely to have received systematic, up-to-date training. This generational effect may be compounded by regional economic development. Evidence from cross-national studies indicates that learners in high-income countries score significantly higher on BPSD-related knowledge and attitudes than their counterparts in other nations (27), consistent with the possibility that regional economic conditions are associated with access to BPSD education. Notably, Xinjiang’s development index has improved steadily in recent years, which may coincide with younger medical personnel having access to better training resources and information access, potentially correlating with better acquisition of BPSD-related knowledge. These hypotheses warrant further investigation in future studies with larger samples and longitudinal designs.

## 5 Limitations and strengths

This study provides an initial assessment of KAP regarding BPSD among medical staff in Xinjiang, helping to fill a knowledge gap in BPSD management in this area and offering baseline evidence for developing targeted training programs tailored to the specific needs of this border ethnic minority region. However, several limitations should be acknowledged. First, in this exploratory study, the use of convenience sampling limited the generalizability of our findings to all medical staff in Xinjiang, and selection bias may be present. Future research should employ random sampling to better represent the situation in Xinjiang. Second, the cross-sectional design cannot establish causal relationships between KAP levels and demographic variables; prospective cohort studies are needed to further validate these associations. Third, we lacked data on the duration and content quality of BPSD-specific training received by participants, which may confound the association between professional background and KAP levels. Fourth, as an exploratory study, we did not perform an *a priori* sample size calculation. However, *post-hoc* power analysis (N = 713) indicated adequate statistical power for the primary analyses (regression: 0.986; group comparison: 0.972). Prospective sample size calculation is recommended for future studies. Additionally, due to the large sample size, although the p-values were significant, the correlation coefficients were very small, suggesting limited practical significance. Based on these limitations, future research should focus on the following areas: 1. exploring the barriers experienced by experienced psychiatric medical staff in updating their knowledge; 2. investigating the mediating role of organizational learning climate in the development of medical staff’s KAP; and 3. conducting intervention studies to design and evaluate customized BPSD training programs for medical staff with different levels of clinical experience.

## Data Availability

The original contributions presented in the study are included in the article/**Supplementary Material**. Further inquiries can be directed to the corresponding author.
