# Supplementary material for: Knowledge, attitude, and practice (KAP) of medical staff toward behavioral and psychological symptoms of dementia (BPSD) in medical institutions in Xinjiang Uygur Autonomous Region
Source: Front Psychiatry. 2026 Jul 17;17:1825862. doi: 10.3389/fpsyt.2026.1825862 (PMC13425138; doi:10.3389/fpsyt.2026.1825862)
Supplement: Supplementary file 2 [file DataSheet2.pdf]

A questionnaire of healthcare workers regarding the knowledge, attitudes, and practices of psychobehavioral symptoms (BPSD) in dementia patients

**Are you familiar with the relevant information about Psychobehavioral Symptoms of Dementia (BPSD)? Knowing = 2 Knowing a little = 1 Not knowing = 0**	
K1	BPSD is a common symptom of dementia, including hallucinations, delusions, anxiety, depression, apathy, personality changes,<br>Behavioral abnormalities and eating and sleeping disorders, etc. ☐ ☐ ☐
K2	BPSD in dementia patients is the primary cause of hospitalization, misdiagnosis, and caregiver distress, and is also the primary target of medication.☐ ☐ ☐
K3	BPSD often interacts with cognitive decline, worsening each other and impacting patients' quality of life.☐ ☐ ☐
K4	BPSD in dementia patients often occurs in infants and young children. ☐ ☐ ☐
K5	BPSD assessment should be performed on all dementia patients. ☐ ☐ ☐
K6	For elderly patients with behavioral abnormalities, it is essential to test their cognitive function, which helps in the early diagnosis and differentiation of<br>dementia. ☐ ☐ ☐<br>[illegible]
K7	BPSD treatment should follow the principle of individualization. ☐ ☐ ☐
K8	The Neuropsychiatric Symptom Questionnaire (NPI) can comprehensively assess multiple types of BPSD and is often used to evaluate the efficacy of<br>medications on psychiatric symptoms. ☐ ☐ ☐
K9	Medications for treating BPSD in dementia mainly include cognitive enhancers, antipsychotics, antidepressants, and mood stabilizers. ☐ ☐ ☐
K10	Non-pharmacological treatments are diverse and have been shown to improve cognitive function in dementia patients and reduce the occurrence of BPSD.<br>☐ ☐ ☐
K11	BPSD is an important factor affecting care burden; empowering caregivers and providing them with relevant support can reduce care burden. ☐ ☐ ☐
K12	Providing caregiving skills training to dementia caregivers is particularly important for improving BPSD in dementia patients. ☐ ☐ ☐
**ttitude toward the appearance of psychobehavioral symptoms (BPSD) in people with dementia? Very positive = 4 Positive = 3 Average = 2 Negative = 1 Very**	
A1	What is your work attitude when a dementia patient experiences BPSD? ☐ ☐ ☐ ☐ ☐
A2	Do you actively communicate with dementia patients to alleviate their BPSD? ☐ ☐ ☐ ☐ ☐
A3	Do you actively choose applicable neuropsychological scales to assess the BPSD of dementia patients? ☐ ☐ ☐ ☐ ☐
A4	When a dementia patient experiences BPSD, do you actively assess the causes of its onset or exacerbation? ☐ ☐ ☐ ☐ ☐
A5	Dementia patients with BPSD require long-term management and follow-up. What is your attitude? ☐ ☐ ☐ ☐ ☐
A6	What is your attitude towards early non-pharmacological intervention for BPSD in dementia patients? ☐ ☐ ☐ ☐ ☐
A7	Are you actively involved in education or training regarding BPSD in dementia patients? ☐ ☐ ☐ ☐ ☐
A8	What is your attitude towards providing relevant support to dementia caregivers? ☐ ☐ ☐ ☐ ☐
**: practice with patients with dementia experiencing psychiatric behavioral symptoms (BPSD). Very positive = 4 Positive = 3 Average = 2 Negative = 1 Very Ne**	
P1	Do you develop personalized follow-up plans for dementia patients with BPSD?<br>And do you inform them and their families of the importance of long-term follow-up? ☐ ☐ ☐ ☐ ☐
P2	I will improve BPSD in the early stages of its onset and treatment.<br>Guide caregivers to conduct individualized non-pharmacological interventions for dementia patients. ☐ ☐ ☐ ☐ ☐
P3	For dementia patients, I would recommend the use of cognitive-promoting drugs by both patients and caregivers.<br>I will also improve BPSD while improving patients' cognitive function. ☐ ☐ ☐ ☐ ☐
P4	I will conduct risk assessments for dementia patients with BPSD, including assessments of emotional symptoms, psychiatric symptoms,<br>Assessment of multiple areas, including disinhibition and impulsive aggressive behavior, sleep, etc. ☐ ☐ ☐ ☐ ☐
P5	I will educate dementia patients with BPSD and their caregivers on the importance of taking medication as prescribed and managing it properly. ☐ ☐ ☐ ☐ ☐
P6	Do you actively conduct care training for dementia patients with BPSD? ☐ ☐ ☐ ☐ ☐
P7	Do you actively follow research progress on BPSD in dementia patients? ☐ ☐ ☐ ☐ ☐
